# 2082. Effectiveness of Bivalent mRNA Vaccines in Preventing SARS-CoV-2 Infection Among Children Aged 5-17 years: an Evaluation of Multicenter Prospective Cohorts, United States, September 2022 - January 2023

**DOI:** 10.1093/ofid/ofad500.152

**Published:** 2023-11-27

**Authors:** Leora R Feldstein, Amadea Britton, Lauren Beacham, Ryan E Wiegand, Jasmine Ruffin, Melissa Briggs-Hagen, Ashley Fowlkes, Jefferey L Burgess, Alberto Caban-Martinez, Helen Y Chu, Janet A Englund, Kurt Hegmann, Zuha Jeddy, Karen Lutrick, Emily T Martin, Jennifer K Meece, Claire Midgley, Arnold Monto, Gabriella Newes-Adeyi, Leah Odame-Bamfo, Lauren E W Olsho, Sharon Saydah, Ning Smith, Laura Steinhardt, Harmony Tyner, Sarang K Yoon, Manjusha Gaglani, Allison L Naleway

**Affiliations:** Centers for Disease Control and Prevention, Atlanta, GA; Centers for Disease Control and Prevention, Atlanta, GA; Centers for Disease Control and Prevention, Atlanta, GA; Centers for Disease Control and Prevention, Atlanta, GA; Centers for Disease Control and Prevention, Atlanta, GA; Centers for Disease Control and Prevention, Atlanta, GA; Centers for Disease Control and Prevention, Atlanta, GA; University of Arizona Zuckerman College of Public Health, Tucson, Arizona; University of Miami, Miller School of Medicine, Miami, Florida; University of Washington, Seattle, Washington; Seattle Children’s Hospital, Seattle, Washington; U.Utah, SLC, Utah; Abt Associates, Atlanta, Georgia; University of Arizona College of Medicine, Tucson, Arizona; University of Michigan, Ann Arbor, MI; Marshfield Clinic Research Institute, Marshfield, Wisconsin; CDC/DDID/NCIRD/DVD; University of Michigan, Ann Arbor, MI; Abt Associates, Atlanta, Georgia; Baylor Scott & White Health Research Institute, Plano, Texas; Abt Associates, Atlanta, Georgia; Centers for Disease Control and Prevention, Atlanta, GA; Kaiser Permanente Center for Health Research, Portland, OR; Centers for Disease Control and Prevention, Atlanta, GA; St. Luke's Regional Healthcare System, Duluth, Minnesota; University of Utah School of Medicine, Salt Lake City, Utah; Baylor Scott & White Health, Temple, TX; Kaiser Permanente Center for Health Research, Portland, OR

## Abstract

**Background:**

The bivalent mRNA COVID-19 vaccine booster dose, composed of mRNA from ancestral and Omicron BA.4/BA.5 strains, was recommended for adolescents aged ≥12 years on September 1, 2022, and for children aged 5–11 years on October 12, 2022. However, data demonstrating the effectiveness of bivalent boosters among children and adolescents are limited. During Omicron variant sublineage predominance, September 4, 2022 – February 4, 2023, we conducted a multicenter prospective cohort study at 7 sites in the United States to assess vaccine effectiveness (VE) of bivalent COVID-19 vaccine boosters against laboratory-confirmed SARS-CoV-2 virus infection among children aged 5–17 years.

**Methods:**

Participants collected weekly nasal swabs, irrespective of symptoms, and at onset of symptoms if present outside of their weekly swab cadence. Vaccination status was captured from periodic surveys (self-report), supplemented with queries from the state immunization information systems, and abstraction of electronic medical records system, when available. All respiratory swabs were tested for SARS-CoV-2 using reverse transcription-polymerase chain reaction. Symptomatic infection was defined as ≥2 COVID-like illness symptoms within 7 days of specimen collection. Cox proportional hazards models were used to estimate hazard ratios of infections comparing participants with receipt of a bivalent booster to participants without (either unvaccinated or received monovalent only), adjusting for age, sex, race/ethnicity, underlying health conditions, prior infection status, geographic site, and local virus prevalence.

**Results:**

Among 3,331 participants aged 5-17 years, adjusted VE against infection was 51% (95% CI: 29–66%). When stratified by age, adjusted VE was 48% (95% CI: 15-68%) for 5-11 year old participants and 55% (95% CI: 17-76%) for 12-17 year old participants. Against symptomatic infection, adjusted VE among 5-17 year old participants was 51% (95% CI: 14-72%).

**Conclusion:**

These results demonstrate that the COVID-19 bivalent booster reduces risk of SARS-CoV-2 infection and symptomatic illness among children and adolescents. All eligible children and adolescents should remain up to date with recommended COVID-19 vaccinations.

**Disclosures:**

**Helen Y. Chu, MD, MPH**, Abbvie: Advisor/Consultant|Ellume: Advisor/Consultant|Ellume: Grant/Research Support|Merck: Advisor/Consultant|Pfizer: Advisor/Consultant|Vir: Advisor/Consultant **Janet A. Englund, MD**, Ark Biopharma: Advisor/Consultant|AstraZeneca: Advisor/Consultant|AstraZeneca: Grant/Research Support|GlaxoSmithKline: Grant/Research Support|Meissa Vaccines: Advisor/Consultant|Merck: Grant/Research Support|Moderna: Advisor/Consultant|Moderna: Grant/Research Support|Pfizer: Advisor/Consultant|Pfizer: Grant/Research Support|Sanofi Pasteur: Advisor/Consultant **Emily T. Martin, PhD, MPH**, Merck: Grant/Research Support **Arnold Monto, MD**, Roche: Advisor/Consultant|Roche: Honoraria

